# Maxon is an Optimal Suture for Bile Duct Anastomoses in Pigs

**DOI:** 10.1155/1993/26569

**Published:** 1993

**Authors:** Phil Jeans, Pauline Hall, Yong-Feng Liu, Robert A. Baker, Andrew Holt, Gino T. P. Saccone, John R. Harvey, Jan Scicchitano, James Toouli

**Affiliations:** 1 Departments of Surgery, Anaesthesia and Intensive Care, & Histopathology Flinders University Bedford Park Adelaide 5042 Australia; 2 Dept Surgery Flinders Medical Centre Bedford Park 5042 Australia

## Abstract

*Background.* Three commonly used sutures were tested in a pig model of bile duct anastomosis to assess their relative contributions to inflammation and scarring.

*Methods.* Thirty pigs were randomised to bile duct division and anastomosis with either polyglyconate (Maxon), polyglactin 910 (Vicryl) or polypropylene (Prolene). Half the animals were sacrificed at two weeks and the remainder at 23 weeks. Anastomoses were assessed by cholangiography, scanning electron microscopy and light microscopy.

*Results.* There was less short term histological reaction with the two monofilament materials, Prolene and Maxon, compared to the braided suture Vicryl. Maxon was associated with less long term inflammation than Prolene, was found to handle better, and has an advantage over Prolene by being absorbable.

*Conclusion.* Maxon is an optimal suture for bile duct anastomoses. Its long term absorption characteristics make it suitable for situations where bile duct healing may be delayed.


*HPB Surgery*, 1993, Vol. 7, pp. 111–124
Reprints available directly from the publisher
Photocopying permitted by license only

© 1993 Harwood Academic Publishers GmbH
Printed in the United States of America

### MAXON IS AN OPTIMAL SUTURE FOR BILE DUCT ANASTOMOSES IN PIGS

PHIL JEANS, PAULINE HALL, YONG-FENG LIU, ROBERT A. BAKER, ANDREW HOLT, GINO T.P. SACCONE, JOHN R. HARVEY, JAN SCICCHITANO and JAMES TOOULI

*Departments of Surgery, Anaesthesia and Intensive Care, & Histopathology, Flinders University, Bedford Park, Adelaide, South Australia, 5042*

*(Received 7 March 1993)*

*Background.* Three commonly used sutures were tested in a pig model of bile duct anastomosis to assess their relative contributions to inflammation and scarring.
*Methods.* Thirty pigs were randomised to bile duct division and anastomosis with either polyglyconate (Maxon), polyglactin 910 (Vicryl) or polypropylene (Prolene). Half the animals were sacrificed at two weeks and the remainder at 23 weeks. Anastomoses were assessed by cholangiography, scanning electron microscopy and light microscopy.
*Results.* There was less short term histological reaction with the two monofilament materials, Prolene and Maxon, compared to the braided suture Vicryl. Maxon was associated with less long term inflammation than Prolene, was found to handle better, and has an advantage over Prolene by being absorbable.
*Conclusion.* Maxon is an optimal suture for bile duct anastomoses. Its long term absorption characteristics make it suitable for situations where bile duct healing may be delayed.

KEY WORDS: Bile duct, Anastomosis, Suture, Pigs

### INTRODUCTION

Operations on the bile ducts are required in a wide range of surgical situations, from retrieval of bile duct stones, traumatic injury and cancer surgery to orthotopic liver transplantation, however the bane of bile duct surgery is the subsequent development of a biliary stricture[1–6]. Closure or reconstruction of the bile ducts is done with sutures however the occasional occurrence of subsequent stricturing and the development of bile duct stones around extruded suture material has meant a search for a suture material which avoids these complications. In one canine study bile duct anastomosis was achieved by using fibrin glue[7], thus eliminating the potential problems associated with suture material, but to our knowledge this technique has not yet been reported in humans.

The ideal material should be non-traumatic to the bile duct wall, incite minimal inflammation and preferably be absorbed over a suitable time frame which avoids anastomotic breakdown in situations where healing may be delayed. The latter

The financial support of Cyanamid Australia Pty. Limited is gratefully acknowledged.

Address correspondence to: Professor James Toouli, Dept Surgery, Flinders Medical Centre, Bedford Park, 5042, Australia situation can arise in trauma (either accidental or iatrogenic) or in surgery where there may be associated catabolism and/or sepsis, or in liver transplantation where immunosuppression and ischaemia reperfusion injury are added to the risks of major surgery.

Available suture materials are associated with one or more shortcomings. In most situations bile duct anastomoses have been done using short term absorbable materials such as chromic catgut, polyglycolic acid (Dexon) or polyglactin-910 (Vicryl). However in liver transplantation, because of the shorter life span of these absorbable sutures when they are exposed to bile, non-absorbable materials such as polypropylene (Prolene) have been used by some groups. The development of monofilament absorbable sutures with a longer tissue life than Vicryl, Dexon or chromic catgut, i.e. polydioxanone (PDS) and polyglyconate (Maxon), presents surgeons with sutures which may meet most of the requirements for operations on the biliary tract, including those where healing may be delayed[8,9].

The aims of this study were to evaluate the integrity of the bile duct anastomosis in a pig model of bile duct division and anastomosis using three types of suture material: Maxon, Vicryl and Prolene.

### METHODS

Thirty large white pigs of both sexes (mean weight 49.2 ± 7.3, range 33–57Kgs) were randomised to have cholecystectomy followed by bile duct division and anastomosis with either Maxon, Vicryl or Prolene so that there were three groups of ten. Each group was then divided into five animals that were sacrificed at two weeks and five animals that were sacrificed at 23 weeks. One pig died during anaesthetic induction and was replaced by a new animal so that altogether 31 pigs were used in the study.

### *Procedure*

Animals were fasted from food for 24 hours but allowed water ad libitum. Following immobilisation in a restraining cage, general anaesthesia was induced by inhalation of halothane via a large face mask. Once anaesthetised, the pigs were rolled onto their back and intubated. General anaesthesia was maintained with halothane inhaled from a circle circuit with a carbon dioxide absorber via an endotracheal tube. An ear vein was cannulated and all animals were given a litre of Hartmann's solution and Gentamycin 80mg i.v. A midline abdominal incision was made and initially the cystic duct and bile duct identified.

The gallbladder was then removed. The peritoneum overlying the supraduodenal bile duct was incised and approximately a 2–3 cm length of bile duct was freed from the surrounding adventitia. Duct diameter in the pig ranged from 4–15mm (mean 9.9 ± 2.9 in the two week sacrifice group).

A transverse division of the duct was made with a scalpel to avoid a crush injury of the ends. The incision was made in the common bile duct below the cystic duct entry. Because of the variability in the level of the cystic duct and bile duct junction the anastomoses were made at a variable level above the duodenum. The mean distance of the anastomoses above the duodenum (measured from the cholangiograms at sacrifice) was 26.5mm (range 12–50) in the two week sacrifice group and 31mm (range 8–70) in the 23 week sacrifice group.

The bile duct anastomosis was then performed using either 5-O(USP) Maxon, Prolene or Vicryl. Generally ten interrupted sutures were placed but in larger ducts as many as 14 sutures were used. The anastomoses were assessed for bile leakage and in several cases this led to an extra suture. The peritoneum was then closed over the anastomosis with a 4-0 Vicryl suture in all but five of the animals. These animals had the peritoneum closed with Maxon or Prolene. No stent or T-tube was used in the duct. The laparotomy incision was closed with continuous 1 Nylon and subcuticular 3-0 Dexon.

On emergence from anaesthesia a single dose of buprenorphine or flunixin, both long acting agents, was usually sufficient for postoperative analgesia. However all animals were assessed for evidence of pain (apathy, hunched back or restricted movement) and a further dose of analgesic given if required.

The animals were observed daily during the first two weeks and every 1–2 weeks after that in the 15 animals to be sacrificed at 23 weeks. These latter animals doubled their weight during the 23 weeks and was consistent with the expected weight gain in animals of this age. All animals were assessed for clinical signs of general well-being and obstructive jaundice.

The anaesthetic induction and maintenance for the second procedure was identical to the first except that no antibiotic was administered. The laparotomy was reopened and adhesions divided to expose the bile duct. The peritoneum was freed from the anastomosis. The duct was then excised "en block" with the duodenum and a liver biopsy was collected. Both ends of the duodenum were ligated and a catheter inserted into the common hepatic duct and tied securely. The duct was placed in oxygenated Krebs solution and transferred to the radiology department.

### *Cholangiography*

All specimens had cholangiography performed using the technique of Sharp[8]. The specimens were placed directly onto the X-ray plate and infused with 30% angiografin at 25cm $H_2O$ pressure as measured by a manometer.

The cholangiograms were used to measure the diameter of the bile duct 0.5cm and 1.5cm above and below the anastomosis and at the anastomosis itself. In cases where the specimen had been cut short or where the anastomosis was closer than 1.5cm to the tapered sphincter segment the largest diameter was used. Using the 1.5cm measurements, the technique of Sharp comparing the ratios of proximal to distal duct diameter (the dilatation ratio) and the anastomosis to distal diameter (the stricture ratio) was calculated[8]. The index used by Klopper to assess anastomotic stricturing was also calculated to compare sutures[10]. The index $= 100 \times 2a/(b + c)$ where $a$ is the diameter of the bile duct 0.5cm proximal to the anastomosis, $b$ is the anastomotic diameter and $c$ is the diameter of the duct 0.5cm distal to the anastomosis.

The sphincter segment was separated from the duct specimen and placed in 10% buffered formalin and the remaining bile duct was then divided longitudinally into two hemi-cylinders which contained the anastomosis. Both hemi-cylinders were washed with normal saline to clear mucus and fibrinous debris from the epithelium and anastomosis. One hemi-cylinder was placed in 10% neutral buffered formalin for light microscopy and the other in 2% glutaraldehyde in phosphate buffer pH 7.4) for fixation for scanning electron microscopy.

*Light Microscopy*

Paraffin sections were prepared and stained with haematoxylin and eosin, and sirius red for collagen. The sections were examined by a single pathologist who was blinded as to which suture was used. A simple grading system was used to judge the following histological features at the bile duct anastomosis; neutrophil polymorph numbers, mononuclear cell numbers, proliferating fibroblasts, collagenous scar tissue, residual suture material, foreign body giant cells and epithelial hyperplasia. The grading system employed a numerical notation, 0,1,2 or 3 for nil, mild, moderate or marked presence of the particular histological feature. Polarised light was used to assess the presence of refractile foreign material i.e. sutures.

The liver biopsy sections were stained with haematoxylin and eosin and examined for evidence of biliary obstruction.

*Scanning Electron Microscopy*

After emersion fixation in glutaraldehyde, the specimens were post-fixed in 1% osmium tetroxide in phosphate buffer, dehydrated through a series of graded ethanol and then critical point dried. The dried specimens were mounted on aluminium stubs and coated with gold in a Polaron E5200 sputter coater. The specimens were then examined in an Etec Autoscan at 20,000 volts. Photographic records were obtained at 15× and 30× magnification using a standard working distance. Anastomoses were then compared particularly for suture presence and epithelial thickening which was measured as the maximum width of the abnormal mucosa.

*Statistical Analysis*

Results have been expressed as mean ± one standard deviation. Anova was used to compare the dilatation and stricture ratios of the different suture materials. P values $<0.05$ were considered statistically significant.

In conjunction with this study we evaluated changes in blood flow in the region of the anastomosis and any alteration in sphincter of Oddi motility following bile duct division. The results of these studies will form the subject of separate reports.

Animal ethics: The study was approved by the animal ethics committees of Flinders Medical Centre and the Commonwealth Scientific and Industrial Research Organisation.

### RESULTS

All animals made a relatively uncomplicated early post-operative recovery. No animal was clinically jaundiced at any stage as judged by scleral, urine and faecal colour. There were several minor wound infections which drained spontaneously and then healed. Animals in the 23 week sacrifice group outgrew the Nylon suture which had been used to close the muscle layer of their abdominal incision. In several of these animals the suture snapped and protruded through the wound at which time the suture was removed.

Two animals died in the long survivor group. The first animal died at 23 days post-operation of an illness which included hypothermia, anaemia and lethargy. At autopsy no source of blood loss was found in the gastrointestinal tract and the biliary surgery was uncomplicated. Several animals from the same piggery had died at about the same time of a similar illness, presumed viral, but never clearly identified.

The second animal died of a small bowel strangulation at two months. At autopsy the biliary surgery was also uncomplicated but extensive adhesions had formed in the abdominal cavity. One of these adhesions produced the bowel obstruction and subsequent strangulation. One animal had been sutured with Vicryl and the other with Prolene leaving only 4 animals in each of those groups for histological and cholangiographic assessment.

### *Cholangiography*

The anastomosis was easily identified on cholangiography in the animals sacrificed at two weeks as a narrowed area. There was no significant proximal dilatation. (See Figure 1A) The dilatation and stricture ratios were similar for all three sutures. (See Tables 1 and 2) When the specimens were opened a large fibrin clot was often found at the anastomosis which had contributed to the apparent narrowing seen on cholangiography.

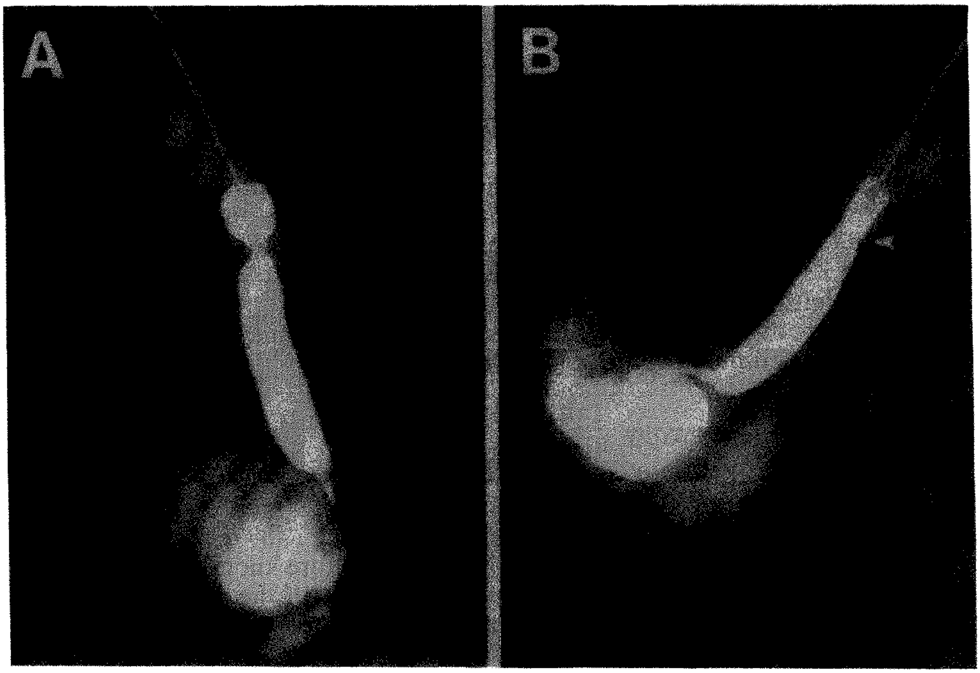

**Figure 1** A: Cholangiogram from an animal sacrificed at 2 weeks showing narrowing at the anastomosis. B: Cholangiogram from an animal sacrificed at 23 weeks. The anastomosis is barely visible as an indentation near the upper end of the duct (arrow).

**Table 1** Dilatation ratios (proximal width to distal width)

| *Survival Time* | *Maxon* | *Suture Type* *Prolene* | *Vicryl* |
|---|---|---|---|
| 2 weeks | .97±.22 | .91±.21 | 1.1±.46 |
| 23 weeks | 1.1±.32 | 1.2±.9 | 1.0±.6 |

Legend: Values represent mean ± one standard deviation.
Ratio >1.0 represents proximal dilatation.

**Table 2** Stricture ratios (anastomotic width to distal width)

| *Survival Time* | *Maxon* | *Suture Type* *Prolene* | *Vicryl* |
|---|---|---|---|
| 2 weeks | .65±.23 | .63±.35 | .76±.30 |
| 23 weeks | 1.1±.8 | 1.1±.7 | .8±.3 |

Legend: Values represent mean ± one standard deviation. Stricture ratio less than 1.0 represents anastomotic narrowing.

In 7 of the 13 animals sacrificed at 23 weeks the site of the anastomosis could not be identified on the cholangiograms. When these ducts were opened and the anastomosis identified visually the cholangiograms were marked to indicate the site of the anastomosis. The dilatation and stricture ratios were similar for all three sutures in the 23 week sacrifice group. One animal in each group had a stricture ratio <0.5 (0.33, 0.38, 0.46). In each of these cases the dilatation ratio was <1.2 indicating that the stenoses had not produced significant obstruction. The luminal diameters ranged from 3–6mm at the anastomoses in these animals and was not obstructive. Figure 1B shows a representative cholangiogram from an animal sacrificed at 23 weeks. The histological features of the anastomoses are described below (vide infra).

When Klopper's index[10] was plotted as a histogram no major differences were seen between the different sutures (Figure 2).

### *Scanning Electron Microscopy*

In the animals sacrificed at two weeks, several features were apparent. All anastomoses were marked by a mucosal thickening (see Figures 3A and 3D) and in many cases the suture had rotated so that the knot which had been placed external to the duct had migrated into the duct lumen. These suture knots formed a surface for the fibrin build up at the anastomosis seen in many of the early cases. Vicryl sutures were sometimes found to be fragmenting at this stage (Figure 3F).

Specimens from this group were examined at 15× and 30× magnification and the width of the mucosal thickening was measured. The least thickening was seen with Maxon whilst Prolene showed the most extensive thickness. The mean widths were 2.6 ± 0.5mm for Maxon, 2.9 ± 0.7mm for Vicryl and 3.0 ± 0.6mm for Prolene. These differences were not statistically significant.

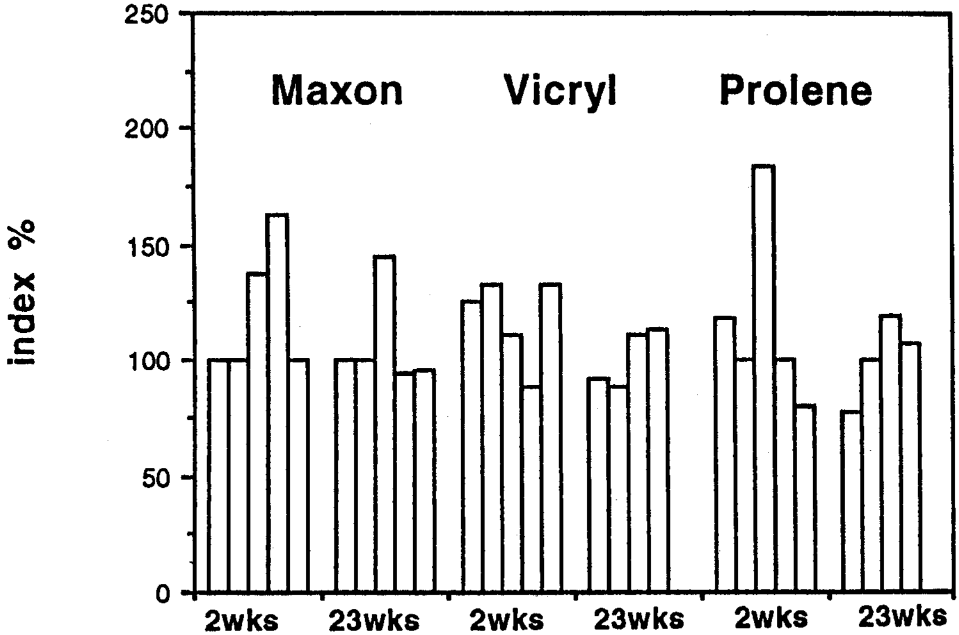

**Figure 2** Anatomical index of the common bile duct for each suture type and each animal (as per Klopper)[10]. A value >100% represents anastomotic narrowing with proximal dilatation.

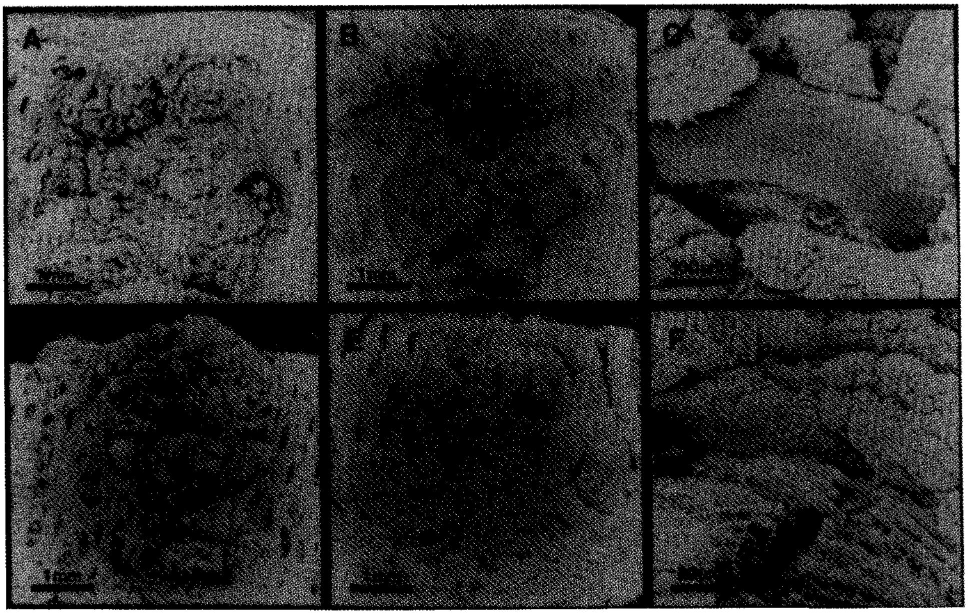

**Figure 3** Scanning electron microscopy views of the anastomoses. A: A vicryl sutured anastomosis at two weeks showing marked mucosal prominence. Compare with D which is a Maxon sutured anastomosis at two weeks. B: A Prolene sutured anastomosis at 23 weeks showing persistence of suture material and rotation of knots into the lumen. C: Maxon at two weeks showing no breakdown. D: A Maxon sutured anastomosis at two weeks showing early rotation of knots into the lumen. E: A Maxon sutured anastomosis at 23 weeks. The anastomosis is barely visible as a ridge without the usual mucosal pits. F: Vicryl at 2 weeks showing fracture and fragmentation.

In the animals sacrificed at 23 weeks the anastomoses were often difficult to recognise except in cases where Prolene sutures remained *in situ*. (Figure 3B) The anastomoses were recognisable as a flattened area displaying reduced numbers of mucosal pits (Figure 3E). In those anastomoses sutured with Prolene, some but not all of the sutures had disappeared, presumably extruded into the lumen of the bile duct and passed into the duodenum.

### *Histological Assessment*

The braided Vicryl was easy to recognise at two weeks but the monofilament sutures, Maxon and Prolene could not be distinguished from one another.

At two weeks the most exuberant inflammatory response was seen with Vicryl sutures. The features included microabscesses, granulomatous inflammation and large numbers of foreign body giant cells. The mean histological score for polymorph numbers was 1.4 compared to only 0.6 for each of the other two sutures. Epithelial hyperplasia (Figure 4) was seen in some animals in association with each of these suture materials but was also more pronounced with Vicryl, the mean score being 2.0 compared to only 1.0 for Maxon (See Table 3).

In some of the anastomoses performed with monofilament sutures there was slightly less fibroblastic proliferation and collagen deposition than with the braided Vicryl. In several animals that had anastomoses sutures with monofilamentous materials but where the overlying peritoneum had been closed with Vicryl, more pronounced inflammation was found adjacent to the anastomosis.

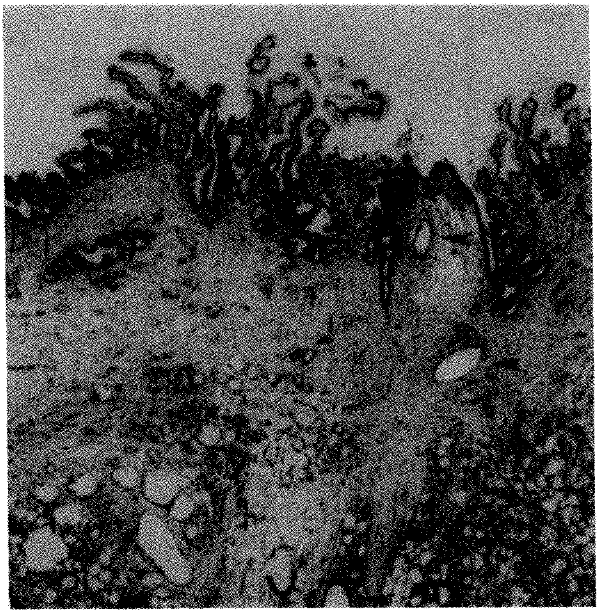

**Figure 4** Histological appearance of a Maxon sutured anastomosis at two weeks. Epithelial hyperplasia is present but is not as prominent as in cases sutured with Vicryl. Several suture holes are present showing minimal surrounding inflammation (arrow). Periductal inflammatory changes are probably asociated with the peritoneal sutures. H&E, original magnification ×20.

**Table 3**

| | Suture | | | | | |
|---|---|---|---|---|---|---|
| | 2 weeks | | | 23 weeks | | |
| | *Maxon* | *Prolene* | *Vicryl* | *Maxon* | *Prolene* | *Vicryl* |
| Histological Feature | | | | | | |
| Polymorphs | .6 | .6 | 1.4 | 0 | 0 | 0 |
| Mononuclear Cells | 1.8 | 2.1 | 2.4 | 0.4 | 1.0 | 0.75 |
| Proliferating Fibroblasts | 2.4 | 2.8 | 3.0 | 0 | 1.0 | 0.5 |
| Collagen 'Scar Tissue' | 2.4 | 2.8 | 3.0 | 1.4 | 2.25 | 2.0 |
| Suture | 1.4 | 2.4 | 2.4 | 0.6 | 1.5 | 0.5 |
| Foreign Body Giant Cells | 1.4 | 1.8 | 2.2 | 0.4 | 0.75 | 0.5 |
| Epithelial Hyperplasia | 1.0 | 1.8 | 2.0 | 0 | 0.25 | 0.5 |
| Mean Histological Score | 11 | 14.3 | 16.4 | 2.8 | 6.75 | 4.75 |

Legend: Histological Assessment — mean score for each histological feature in 2wk and 23wk sacrifice groups (0 = none, 1 = mild, 2 = moderate, 3 = marked).

At 23 weeks, in those cases where suture material persisted there was usually a foreign body giant cell reaction, a mild mononuclear cell infiltrate, mild fibroblastic proliferation and rather more mature fibrous tissue than in cases where the sutures had been completely absorbed. Distinct differences between the non-absorbable Prolene and the two absorbable sutures were thus apparent. Histological scores for mononuclear cell numbers, proliferating fibroblasts, collagen and foreign body giant cells were all higher for Prolene compared to the absorbable sutures (see Table 3). Of the four animals identified as having the smallest amount of residual scarring at the anastomosis three had been sutured with Maxon and one with Vicryl.

In the animals with a stricture ratio $<0.5$ further histological sections were reexamined to see if any contributing factor could be identified. Excessive scar tissue or collagen was not found and it remained unclear as to why these anastomoses had more narrowing than the others.

The liver biopsies from all animals did not show any features to suggest extrahepatic biliary obstruction, in particular there was no evidence of portal tract oedema, fibrosis, inflammation, bile ductular proliferation or bile stasis.

### *Handling Characteristics*

The surgeon performing the anastomoses found that the Maxon had the best handling characteristics in that it slid through the tissue freely and was pliable enough to knot well. Vicryl had a tendency to saw the tissue whilst Prolene's better "memory" made it slightly more difficult to tie knots without traumatising the tissue. These assessments are subjective but certainly correspond to the known physical characteristics of the sutures.

### DISCUSSION

The pathogenesis of biliary strictures that follow bile duct anastomoses remains an important unsolved riddle although it is suspected that ischaemia, tension and bacterial infection play a critical role[11,12,13]. In performing anastomoses of bile ducts, surgeons are always concerned that an excessive inflammatory response may assist in stricture formation. For these reasons, the choice of suture material for biliary anastomoses is important.

The ideal suture material should be relatively inert, subject to eventual tissue digestion, yet provide sufficient strength to enable healing to occur, even when healing may be delayed such as in liver transplantation where a patient has the added risks of rejection, immunosuppression, ischaemia-reperfusion injury and sepsis. At the same time it is desirable that the suture material does not act as a potential nidus for stone formation.

Of the sutures that are currently in popular use, each has its own limitations. Chromic catgut has a reduced half-life when exposed to bile and excites an intense inflammatory tissue reaction as it is digested by the process of proteolysis[14].

The absorbable sutures with a slightly longer half-life, Dexon and Vicryl, are less affected by exposure to bile but because they are braided to overcome the rigidity that exists in their monofilament form, they have the potential of inciting a more intense inflammatory response than would be expected from a monofilament suture[14]. They also suffer from a rapid loss of strength after seven days[15].

Whilst non-absorbable monofilament sutures such as Prolene overcome the problem of a short half-life, and whilst relatively inert, they do provide a focus for possible stone formation and an ongoing inflammatory response.

The emergence of monofilament absorbable sutures with a longer half life, such as Maxon and PDS, offers surgeons the potential of an ideal suture material for bile duct anastomoses[9,16]. The major difference between these two sutures is in the handling characteristics as PDS is 60% stiffer than Maxon[17]. It has also been claimed that as a result of this stiffness that knot security is not as great with PDS[18,19].

In designing this comparison study of Maxon, Vicryl and Prolene, two factors were judged as being of paramount importance. Firstly the sutures needed to be assessed as to stricture potential. Although most biliary strictures develop within weeks or months of the initial surgery they can occur many years later. Way found that approximately 60% of cases presented within 30 days whilst Braasch claimed that the incidence curve does not plateau until 2.5–3 years post-operatively[2,3].

It was deemed important to have a group of animals survive longterm to optimise this stricture potential. In a previous study using Maxon the potential of Maxon to form strictures was studied for only 7 weeks[8]. The timing for examining the anastomoses was also based on previous reports[20,21] which showed that complete healing can be expected at two weeks. Histological grading of the anastomoses provided the most important measure of healing and reaction to suture material. Furthermore, cholangiography provided an objective measure of stricture formation and alteration of bile duct diameter.

Tensiometry measurements were not performed in this study as they were thought to be a poor indicator of an optimal healing response in the bile duct. Although other studies of sutured bile duct anastomoses have used tensiometry as a guide to healing[8,20] we have concerns regarding interpretation of this measurement. Is a strong bile duct anastomosis an indication of good healing or of excessive healing with stricturing as a possible consequence? As the bile duct is not a structure which requires excessive strength to perform its role in our view the strength of an anastomosis is of minor functional significance.

This study has shown that all three sutures tested were satisfactory for performing bile duct anastomoses in healthy animals. This finding has been noted previously in a study which evaluated the degree of stricture formation in pig bile ducts[20]. In this animal species, the bile duct is well vascularised, so problems of ischaemia are not an important factor, hence making this model ideal for comparison of suture material. The cholangiographic stricture and dilatation ratios were not significantly different for the three sutures in either the short or long term. At two weeks the cholangiograms indicated a relative stenosis at the anastomosis, but no proximal dilatation of the duct. Visual inspection of the opened ducts showed that the narrowing was due to mucosal prominence and fibrin build-up at the anastomosis. At 23 weeks the cholangiograms had displayed minimal evidence of stricturing, hence the effect recorded at two weeks is not thought to have adversely influenced the results.

Light microscopy showed that the braided material Vicryl incited the strongest inflammatory response at two weeks and it had the highest histological scores, however once the material was absorbed this response subsided. The non-absorbable suture Prolene had less of an inflammatory response at two weeks but its persistence in the duct wall meant that an inflammatory response was still present at 23 weeks. Maxon showed the least inflammatory reaction at both 2 weeks and 23 weeks. This is a similar finding to the studies of Chiu and Stillman which compared Maxon and Prolene in aortic anastomoses in pigs[22,23].

The finding that the sutures used to close the overlying peritoneum could cause inflammation and fibrosis around the duct and anastomosis suggests that such manoeuvres (designed to restrict bile leakage) should be resisted as they are potentially counter-productive. In this study the resultant inflammation may have influenced the histological assessment even though nearly all animals had the peritoneum closed with Vicryl.

The finding of epithelial hyperplasia on light microscopy and a corresponding mucosal thickening on SEM is an unexpected finding. In previously reported animal studies of healing in bile ducts, the epithelial migration and proliferation usually ceases once epithelial continuity is restored[24,25,26,27]. In our study epithelial hyperplasia was marked at two weeks but had subsided at 23 weeks. The epithelial hyperplasia may be a response to the suture material. It appeared more pronounced with Vicryl as opposed to other sutures. The epithelial hyperplasia was no longer apparent by light microscopy at 23 weeks even in those animals where suture material remained (the Prolene group), however there appeared to be residual foci of it around sutures on scanning electron microscopy (Figure 3B). Another possible factor for epithelial hyperplasia is a secondary response to injury and repair and the release of a variety of growth factors and cytokines by the various cells that are involved in wound healing. There is evidence from embryological studies and duct growth studies that the underlying connective tissue may stimulate ductal epithelial differentiation and growth[28,29,30,31]. This hypothesis is further reinforced by the finding that the epithelial hyperplasia was greatest in those cases where a greater inflammatory and fibrous tissue response was stimulated by the suture material, particularly those sutured with Vicryl.

Surgeons often assess suture materials according to their "ease of handling" during an operation. It was our view that subjectively Maxon was easier to work with than either Vicryl or Prolene. However we do accept that this is a subjective evaluation which varies from surgeon to surgeon.

Our study however has shown that Maxon is potentially a superior suture for use in bile duct anastomoses when compared to Vicryl and Prolene. Although there was no difference found in the potential for stricture formation between the three sutures, Maxon came closest to fulfilling the criteria for an ideal suture — it was associated with the least inflammatory response short term, and long term it degraded completely not leaving remnants which might potentially form the nidus for stone formation.

### *Acknowledgements*

Dr Yong Feng Liu was a post-doctoral research fellow (Dept of Surgery, First University Hospital, China Medical University, Shenyang, China). The Commonwealth Scientific and Industrial Research Organisation and the staff of its Glenthorne site are thanked for the use of their facilities and assistance. John Harvey was funded by a grant from the National Health and Medical Research Council.

### *References*

1. Warren, K.W., Mountain, J.C. and Midell, A.I. (1971) Management of strictures of the biliary tract. *Surg. Clin. Am.*, **51**, 711–731
2. Braasch, J.W. (1985) Postoperative strictures of the bile duct.In: Schwartz, S.I., Ellis, H. (ed.) *Maingot's Abdominal Operations*, 8th ed. Norwalk: Appleton-Century-Crofts, 1951–1978
3. Way, L.W., Bernhoft, R.A. and Thomas, M.J. (1981) Biliary stricture. *Surg. Clin. N. Am.*, **61**, 963–972
4. Moosa, A.R., Mayer, A.D. and Stabile, B. (1990) Iatrogenic injury to the bile duct. *Arch. Surg.*, **125**, 1028–1031
5. Bismuth, H. (1982) Postoperative strictures of the bile duct. In: Blumgart, L.H. (ed.) *The biliary tract*. Ist ed. Edinburgh: Churchill Livingstone, pp.209–218
6. Blumgart, L.H., Kelley, C.J. and Benjamin, I.S. (1984) Benign bile duct stricture following cholecystectomy: critical factors in management. *Br. J. Surg.*, **71**, 836–843
7. Kram, H.B., Garces, M.A., Klein, S.R. and Shoemaker,W.C. (1985) Common bile duct anastomosis using fibrin glue. *Arch. Surg.*, **120**, 1250–1256
8. Sharp, K.W., Ross, C.B., Tillman, V.N. and Dunn, J.F. (1989) Commqn Bile Duct Healing: Do different absorbable sutures affect stricture formation and tensile strength? *Arch. Surg.*, **124**, 408–414
9. Hoile, R.W. (1983) The use of a new suture material (Polydioxanone) in the biliary tract. *Ann. R. Coll. Surg. Engl.*, **65**, 168–171
10. Klopper,P.J., Kelly, K.A. and Vos,A. (1968) Experimental anastomoses of the common bile duct. *Surgery* **63**, 459–463
11. Rozga, J., Ahrén, B., Andersson, R., Emody, L., Wadström, T. and Bengmark, S., (1991) Effect of biliary infection on common bile duct healing in the rat. *Br. J. Surg.*, **78**, 1329–1331

12. Casey, W.J. and Peacock, E.E., Jr. (1977) Some factors affecting pathophysiology of bile duct stenosis. *Surg. Forum*, **28**, 414–416
13. Northover, J. and Terblanche, J. (1978) Bile duct blood supply: Its importance in human liver transplantation. *Transplantation*, **26**, 67–69
14. Sugimachi, K., Sufian, S., Weiss, M.J., Pavlides, C.A. and Matsumoto, T. (1978) Evaluation of absorbable suture materials in biliary tract surgery. *Int. Surg.*, **63**, 135–139
15. Rodeheaver, G.T., Thacker, J.G. and Edlich, R.F. Mechanical performance of polyglycolic acid and polyglactin 910 synthetic absorbable sutures. *Surg.Gynecol. Obstet.*, **153**, 497–507
16. Katz, A.R., Mukherjee,D.P., Kaganov, A.L. and Gordon, S. (1985) A new synthetic monofilament absorbable suture made from polytrimethylene carbonate. *Surg. Gynecol.Obstet.*, **161**, 213–222
17. Rodeheaver, G.T., Powell, T.A., Thacker, J.G. and Edlich, R.F. (1987) Mechanical performance of monofilament synthetic absorbable sutures. *Am. J. Surg.*, **154**, 544–547
18. Thiede, A., Stüwe, W. and Lünstedt, B. (1985) Vergleich von physikalischen Parametern und Handbungseigenschaften kurzfristig und mittelfristig absorbierbarer Nahtmaterialien. *Chirurg.*, **56**, 803–808
19. Trimbos, J.B., Van Rijssel, E.J.C. and Klopper, P.J. (1986) Performance of sliding knots in monofilament and multifilament suture material. *Obstet. Gynecol.*, **68**, 425–430
20. Northover, J.M.A., Hickman, R. Watson, R.G.K. and Terblanche, J. (1985) Healing of the bile duct anastomosis after transverse choledochotomy or transplantation of the liver in the pig. *Surg. Gynecol. Obstet.*, **160**, 33–36
21. Lamesch, A.J. and Dociu, N. (1986) Microsurgical reconstruction of the biliary duct: experimental study in rats and dogs. *Microsurgery*, **7**, 46–52
22. Chiu, I., Hung, C., Chao, S. and Huang, S. (1988) Growth of the aortic anastomosis in pigs. *J. Thorac.Cardiovasc. Surg.*, **95**, 112–118
23. Stillman, R.M. and Sophie, Z. (1985) Repair of growing vessels: continuous absorbable or interrupted nonabsorbable suture? *Arch. Surg.*, **120**, 1281–1283
24. Simert, G., Hammar, E., Hansson, J.A., Hägerstrand, I. and Vang, J. (1976) Enzyme histochemistry studies of transverse and longitudinal incisions in the common bile duct in rabbits. *Acta Chir.Scand.*, **142**, 527–532
25. Chou, S.T. and Chow, C.W. (1973) A histochemical study of epithelial repair of the common bile duct in rats following mechanical injury. *Pathology*, **5**, 149–161
26. Van Hattum, A.H., James, J., Klopper, P.J. and Muller, J.H. (1979) A model for the study of epithelial migration in wound healing. *Virchows Arch*, **30**, 221–230
27. Van Hattum, A.H., James, J., Klopper, P.J. and Muller, J.H. (1981) Healing of a superficial lesion in the common bile duct of the rabbit. *Neth. J. Surg.*, **33**, 25–31
28. Elias, H. (1967) Recruitment in human bile duct formation. *Acta Hepatosplen.*, **14**, 253–260
29. Shiojiri, N. (1984) The origin of intrahepatic bile duct cells in the mouse. *J. Embryol. Exp. Morph.*, **79**, 25–39
30. Isseroff, H., Sawma, J.T. and Reino, D. (1977) Fascioliasis: Role of proline in bile duct hyperplasia. *Science*, **198**, 1157–1159
31. Vacanti, J.P. and Folkman, J. (1979) Bile duct enlargement by infusion of L-proline: Potential significance in biliary atresia. *J. Pediatr. Surg.*, **14**, 814–818

*(Accepted by S.Bengmark 7 March 1993)*

### INVITED COMMENTARY

An animal model of use for studying the variables of bile duct stricture repair of primary anastomosis has many inherent pitfalls, which include species and anatomic differences. Other problems in this report include a variation as far as distance from the duodenum where the section and anastomosis of the bile duct was made. It has been shown by Northover and Terblanche[1] that in the human bile duct there is variation as to the number of arteries supplying the duct at different levels of the duct, with the common hepatic duct receiving anatomically more arterial blood vessels than the mid portion.

In addition a length of duct, which varied between 2 to 3 cm., was stripped in order to section and then anastomose the duct. Clinically one should strive to have very little stripping of the proximal duct for the anastomosis for fear of depriving that portion of an arterial supply. Ischemic necrosis is a real possibility for the development of subsequent fibrosis and stricturing. These are but some of the variables in this paper, which might explain a relatively wide scatter of data, which impedes a clear definition of results.

In spite of these inherent problems, the authors have clearly reported a difference in pigs between three sutures relative to histologic and scanning examinations of the anastomoses at two weeks and at twenty-three weeks. Whether these differences are relevant to the recurrence of strictures is unknown. It is possible that it might have nothing to do with recurrent stricture and that untoward outcome might be due to some other factor.

In the past, refinements of surgical technique and patient care have reduced the recurrent stricture proportion from 40 per cent to 15 per cent. It is conceivable that the last 15 per cent reduction to perfection might be unattainable.

John W. Braasch, M.D.

*Reference*

1. Northover, J.M.A. and Terblanche, J. (1979) A new look at the arterial supply of the bile duct in man and its surgical implications. *Br. J. Surg.*, **66**, 379–384

